# Dentition of Children at the Breast

**Published:** 1842-09

**Authors:** 


*Dentition of Children at the Breast.* By Professor Trousseau.—Suspecting that the generally received ideas on the dentition of children were not correct, M. Trousseau, from statistical observations not very numerous, has come to conclusions slightly different from the opinions generally believed.

*Period at which the first tooth appears.* This has been determined in 25 children; 13 boys, and 12 girls. It appeared as follows:

| | | | |
|---|---|---|---|
| In 2 boys | at 3 months. | In 1 girl | at 3 months. |
| 2 | at 4 | 2 | at 3 |
| 2 | at 5 | 1 | at 4 |
| 2 | at 6 | 1 | at 5 |
| 2 | at 10 | 3 | at 6 |
| 2 | at 11 | 1 | at 7 |
| 2 | at 14 | 3 | at 9 |
| | | 1 | at 14 |
| Extremes, 3 months, 14 months. | | 2 months, 14 months. | |
| Average, 7 months. | | 6 months. | |

The average time, then, at which the first tooth appears is, from these results, six months and a half; while the general belief at present is, that at eight months it makes its appearance. The common opinion that little girls are more precocious than boys, is also corroborated, as we find a difference of a month between the appearance of the first tooth in boys and in girls.

The first tooth is in general one of the middle inferior incisors. Of twenty-eight children this was the case in twenty-five. In two, a middle superior incisor appeared first; and in a little girl, the first molars preceded all the others.

*Period at which the second tooth appears.* The twenty-five children in whom a middle inferior incisor first showed, had for their second tooth, the other middle inferior incisor. The common opinion is, that this second tooth appears almost at the same time as the first; and it is quite correct. In twenty-five children, the time that elapsed between the first and second tooth, was, 1 day in 4 children; 2 days in 2 children; 3 days in 1 child; 8 days in 3 children; 15 days in 7 children; 30 days in one child; 90 days in 1 child. In six the time could not be determined by the mother.

The middle superior incisors appear next after the two middle inferior. In eighteen children, fifteen had these teeth after the middle inferior incisors. The time that elapses generally between the cutting of the inferior and superior incisors, is more variable than is generally supposed. In thirteen children in whom M. Trousseau was able to verify this; there was a lapse of 8 days in 2 children: 1 month in 3 children; 2 months in 3 children; 3 months in 1 child; 4 months in 1 child; 5 months in 2 children; 10 months in 1 child.

In general, then, there is a considerable interval between the appearance of the second first and the second second teeth. It may be established as a general rule, almost without any exception, that after the appearance of these four teeth, the two lateral superior incisors next cut the gum, and then the two inferior ones, so that the child has only two teeth in the lower jaw, while it has four in the upper. It is curious, M. Trousseau says, that this fact, so well known to mothers who have brought up several children, should be unknown to those who have written specially on the subject. Whilst a considerable period takes place between the first appearance of the inferior and superior incisors, the four superior incisors follow each other at a short interval. After these six teeth have come through, some time elapses before the next, which are generally believed to be the two lateral inferior incisors, appear. M. Trousseau, however, says, that as a general rule, one or several molars, and sometimes even the canine, are protruded before them. After the cutting of the four molars, and the two inferior lateral incisors, another considerable interval of time elapses, when the four canine successively appear; and then after another period, at the age of from twenty-four to thirty months, rarely sooner, the second set of molars come out.

In the second part of his work, M. Trousseau treats of the diseases incidental to teething, and especially of the diarrhœa accompanying it, and he combats the opinion so prevalent, that nothing ought to be done for it. He shows that it is not only not useful, but decidedly injurious to the little patients—that it weakens them, and lays the foundation of marasmus, which if not checked in time, soon goes on to a fatal termination.—*Lond. and Edin. Monthly Jour. Med. Sci.*, May, 1842, from *Gaz. Méd de Paris*, 4th Feb. 1842.

